# X ray screening at entry and systematic screening for the control of tuberculosis in a highly endemic prison

**DOI:** 10.1186/1471-2458-13-983

**Published:** 2013-10-20

**Authors:** Alexandra Sanchez, Veronique Massari, Germano Gerhardt, Ana Beatriz Espinola, Mahinda Siriwardana, Luiz Antonio B Camacho, Bernard Larouzé

**Affiliations:** 1 Programa de Controle de Tuberculose e, Coordenação de Gestão em Saúde Penitenciária, Secretaria de Estado de Administração Penitenciária, Rio de Janeiro, Brasil; 2 INSERM U707, F-75012, Paris, France; 3 UPMC UMR-S707, F-75012, Paris, France; 4 Fundação Athaulfo de Paiva, Rio de Janeiro, Brasil; 5 Departamento de Epidemiologia e Metodos Quantitativos em Saúde, Escola Nacional de Saude Publica, Fiocruz, Rio de Janeiro, Brasil

**Keywords:** Tuberculosis control, X-ray screening, Prisons, Brazil, Incidence

## Abstract

**Background:**

Tuberculosis (TB) is a major issue in prisons of low and middle income countries where TB incidence rates are much higher in prison populations as compared with the general population. In the Rio de Janeiro (RJ) State prison system, the TB control program is limited to passive case-finding and supervised short duration treatment. The aim of this study was to measure the impact of X-ray screening at entry associated with systematic screening on the prevalence and incidence of active TB.

**Methods:**

We followed up for 2 years a RJ State prison for adult males (1429 inmates at the beginning of the study) and performed, in addition to passive case-finding, 1) two “cross-sectional” X-ray systematic screenings: the first at the beginning of the study period and the second 13 months later; 2) X-ray screening of inmates entering the prison during the 2 year study period. Bacteriological examinations were performed in inmates presenting any pulmonary, pleural or mediastinal X-ray abnormality or spontaneously attending the prison clinic for symptoms suggestive of TB.

**Results:**

Overall, 4326 X-rays were performed and 246 TB cases were identified. Prevalence among entering inmates remained similar during 1^st^ and the 2^nd^ year of the study: 2.8% (21/754) and 2.9% (28/954) respectively, whereas prevalence decreased from 6.0% (83/1374) to 2.8% (35/1244) between 1^st^ and 2^nd^ systematic screenings (p < 0.0001). Incidence rates of cases identified by passive case-finding decreased from 42 to 19 per 1000 person-years between the 1^st^ and the 2^nd^ year (p < 0.0001). Cases identified by screenings were less likely to be bacteriologically confirmed as compared with cases identified by passive-case finding.

**Conclusions:**

The strategy investigated, which seems highly effective, should be considered in highly endemic confined settings such as prisons.

## Background

Worldwide, tuberculosis (TB) is an important problem in prison populations, especially in low and middle income countries [1]. In the Rio de Janeiro (RJ) State prison system, the TB incidence rate during the year previous to our study [2] was 2676 per 100,000, 33 times higher compared to the incidence rate of the general population [3], and a 4.6% prevalence was observed in a X-ray systematic screening performed in one of the 32 prison units of the system [4].

In most prisons of low and middle income countries, the TB control program is limited to the diagnosis of cases among inmates attending spontaneously the prison clinic for symptoms suggestive of TB (passive case-finding) and supervised short duration treatment using, in Brazil, the 2HRZ/4HR regimen as recommended by the Ministry of Health [5]. This strategy should remain the priority but should be associated, in highly endemic prisons, with systematic screening as recommended [1,5-9] but seldom applied.

To support this recommendation, we previously developed a mathematical model of TB epidemiology in a highly endemic prison [10] based on data obtained from previous studies [4] and on data extracted from the medical literature. Then, we used this model to simulate the impact of various control strategies. The predictions we obtained showed that the most effective strategy to obtain a rapid and sustained reduction of active TB prevalence in this setting associates basic control methods (passive case-finding and treatment), X-ray screening at entry in prison and annual systematic screenings.

As the impact of this strategy on TB incidence and prevalence in highly endemic confined settings has not been previously studied, we applied this strategy and measured its impact in a prison unit of high TB incidence which we followed up for two years.

## Methods

### Study population

The prison unit which we followed up from 2005 to 2007 is a median security unit for males over 18 years old with a high level of TB endemicity (2004 TB incidence rate: 8686/100,000) [2]. This unit hosted 1429 inmates at the beginning of our study in 22 cells poorly ventilated and overcrowded.

### Intervention strategy and diagnostic criteria

During our 2 year-long study (2005–2007), we implemented the following TB control strategy which associated three components:

1) Two “cross-sectional” X-ray systematic screenings: the first at the beginning of the study period (June 2005), and the second 13 months later (July 2006). Each systematic screening was carried out within six weeks;

2) X-ray screening of inmates entering the prison within one week after admission during the study period from remand centres or other prisons;

3) In addition to the screenings, passive case-finding of TB cases among inmates spontaneously attending the prison clinic during the study period for symptoms suggestive of TB.

Given the design of the study, all inmates investigated during the 2d systematic screening had been investigated during the 1^st^ screening or during the screening at entry.

As previously described [11], after giving his informed consent, each inmate was interviewed face to face using a standardized questionnaire and, regardless of symptoms, underwent, in an X-ray mobile unit, a frontal digital chest X-ray. X-rays were read by AS and, subsequently and independently, by GG without knowledge of AS evaluation. X-ray abnormalities were recorded in a standardized form. Bacteriological examinations were performed in inmates presenting any pulmonary, pleural or mediastinal X-ray abnormality or spontaneously attending the prison clinic for symptoms suggestive of TB. At least two sputum samples were collected from these TB suspects on different days for microscopic sputum smear examination for acid-fast bacteria (AFB) after Ziehl-Neelsen staining. All sputum samples were cultured on Löwenstein-Jensen medium. *Mycobacterium tuberculosis* (*MTB*) isolates were tested for sensitivity to rifampicin, isoniazid (INH) and streptomycin by the proportion method.

Diagnostic criteria for TB cases were as follow [11,12]:

1. Bacteriologically positive cases: subjects with two AFB-positive sputum sample by direct microscopic examination, or one AFB-positive sputum sample with X-ray abnormalities consistent with active pulmonary TB, or positive culture for *MTB*.

2. Bacteriologically negative cases: a) For subjects with negative sputum smear examination and culture, but with presence of patchy infiltration(s) in the upper half of the lungs with or without cavitation and, for those subjects with respiratory or systemic symptoms, absence of radiological improvement after a 10-day course of amoxicillin, the diagnosis of TB was based on radiological and clinical improvement after six months of TB treatment; b) In inmates previously treated for TB and who showed deterioration of their radiographic abnormalities relative to a previous X-ray virgula the diagnosis of new TB was based on radiological and clinical improvement after six months of TB treatment.

HIV serological test was offered to TB cases with pre-and post-test counselling according to the recommendations of the National AIDS Program. TB cases and inmates infected with HIV were treated and followed up according to the recommendations of the Brazilian Ministry of Health [5].

### Statistical analysis

Data were recorded using Excel software and analysed using SAS software version 9.3 for Windows (SAS Institute Inc., Cary, NC). The prevalence of new TB cases identified by screening was expressed as the percentage of cases detected among the total number of inmates screened. The entry and exit times were obtained from the data base of penal records of the RJ State Prison Administration. The incidence rates of TB cases were expressed as number of cases per person-years. The χ^2^ or Fisher’s exact test were used to compare categorical variables, and the Student’s t-test or Anova test for continuous variables. Differences were considered significant if p < 0.05. All tests of significance were two sided.

The protocol was approved by the ethics committee of the National School of Public Health, Oswaldo Cruz Foundation, Rio de Janeiro.

## Results

A total of 4326 X-rays were performed: 1374 and 1244 during the initial and second systematic screenings, 754 at entry in the prison during the first year of study and 954 during the second year. These 4326 X-rays represents 97.7% of the total number of X-rays expected to be performed based on the nominal lists of inmates provided by the prison administration.

Characteristics of inmates examined during the 1^st^ systematic screening and screening at entry during the 1^st^ and 2d year respectively are shown in Table 1. Inmates screened during the 2d systematic screening were excluded from the comparison as they had been already examined during the 1^st^ systematic screening (n = 1374) or at entry (n total = 1708). Inmates in the first systematic screening and those screened at entry had similar characteristics: they were young adults and, on average, had poor schooling, substantial illiteracy rates and lived mostly in poor sections of RJ city and metropolitan area. Many of them had been previously incarcerated and more than ten per cent reported a previous treatment for TB. The mean total time in jail was significantly longer among participants in the systematic screening.

**Table 1:** Characteristics of inmates screened for tuberculosis in a highly endemic prison

	**First systematic screening**	**Screening at entry (first year)**	**Screening at entry (second year)**	**p**
Number	1374	754	954	
Mean age (range)	26.7 (18–63)	26.1 (18–60)	26.3 (18–69)	.06
School years (mean ± sd)	5.3 ± 2.7	5.4 ± 2.7	5.4 ± 2.7	.25
Illiteracy	118/1369 (8.6%)	53/745 (7.1%)	96/952 (10.1%)	.10
Living in favela*	842/1362 (61.8%)	462/754 (61.3%)	552/954 (57.9%)	.19
History of incarceration	533/1372 (38.8%)	300/754 (39.8%)	393/954 (41.2%)	.57
Total time in jail in months (mean ± sd)	40.4 ± 33.9	32.1 ± 38.8	31.0 ± 34.8	<.0001
History of TB treatment	152/1327 (11.4%)	74/728 (10.2%)	99/954 (10.4%)	.25

*Settlement with high density of population and partial urbanization.

For technical reasons, drug susceptibility testing was not systematically performed in TB cases diagnosed during the second year of study, but results were available for 89/127 (70.1%) culture-positive cases diagnosed during the first year of study. One case was resistant to RFM and INH (MDR) and five others were resistant only to INH (n = 2) or SM (n = 3). Another case, initially resistant to INH, was identified as being MDR 6 months later.

### Impact of the TB control strategies implemented

During the study period, a total of 246 TB cases were detected, including 167 cases identified during screenings (118 in systematic screenings and 49 in screening at entry) and 79 cases identified by passive case-finding, i.e. independently of screenings. In addition, community acquired pneumonia was diagnosed in 13 inmates based on clinical and radiological responses to treatment by amoxicillin.

During the initial systematic screening (1374 inmates screened), 83 cases of TB (prevalence: 6.0%) were identified. As shown in Table 2, this prevalence was higher (p < .00001) than that observed during the second systematic screening (2.8%). The prevalences among inmates entering the prison during the first and second year of the study were close (2.8 versus 2.9%). The incidence rate of cases identified by passive case-finding decreased from 42 to 19 per 1000 person-years between the 1^st^ and the 2^nd^ year (p < .0001).

**Table 2:** Prevalence of tuberculosis cases identified by systematic screening and screening at entry in a highly endemic prison

	**Nb TB cases /Nb inmates screened**	**Prevalence (%)**	**p**
Initial systematic screening	83/1374	6.0	<.00001
Systematic screening after one year	35/1244	2.8	
Screening at entry (first year)	21/754	2.8	.98
Screening at entry (second year)	28/954	2.9	

### Characteristics of TB cases

The characteristics of the 246 TB cases diagnosed during the study period are shown in Table 3. As compared with cases identified by screenings, cases identified by passive case-finding were more likely to be bacteriologically confirmed as were the cases with extensive lesions versus other cases (82.3%, 84/102 vs 63.9%, 92/144, p = 0.002).

**Table 3:** Characteristics of tuberculosis cases according to mode of identification in a highly endemic prison

	**Systematic screening**	**Screening at entry**	**Passive detection**	**p**
**Anamnestic variables**				
History of incarceration	54/118 (45.8%)	28/49 (57.1%)	32/79 (40.5%)	.18
Total duration in jail*	48.5 (35.8)	41.0 (35.0)	42.0 (31.8)	.30
History of TB treatment	27/118 (22.9%)	9/49 (18.4%)	22/79 (27.8%)	.48
**Clinical and radiological variables**				
At least one symptom	65/118 (55.1%)	27/49 (55.1%)	69/73 (94.5%)	.00001
Cough ≥ 3 weeks	39/117 (33.3%)	15/48 (31.2%)	41/73 (56.2%)	.0005
Fever	32/118 (27.1%)	11/49 (22.4%)	35/73 (47.9%)	.0004
Weight loss	40/118 (33.9%)	14/49 (28.6%)	19/73 (26.0%)	.83
Thoracic pain	34/118 (28.8%)	15/49 (30.6%)	44/73 (60.3%)	.00001
Night swears	19/118 (16.1%)	9/49 (18.4%)	15/73 (20.5%)	0.008
Cavity	26/118 (22.0%)	11/49 (22.4%)	27/79 (34.2%)	.14
Extensive lesions**	45/118 (38.1%)	22/49 (44.9%)	35/79 (44.3%)	.61
HIV seropositive	3/105 (2.9%)	1/27 (3.7%)	0/24	.75
**Bacteriological variables**				
Smear (+)	57/118 (48.3%)	25/49 (51.0%)	60/79 (76.0%)	.0004
Smear (−) / Culture (+)	23/118 (19.5%)	4/49 (8.2%)	8/79 (10.1%)	
Smear (−) / Culture (−)	38/118 (32.2%)	20/49 (40.8%)	11/79 (13.9%)	

*Mean (s.d.) in months; **Presence of bilateral lesions and/or cavity.

The 167 cases identified by systematic screening and screening at entry had similar anamnestic, clinical and bacteriological characteristics. In particular, one half of them declared at least one symptom, one third declared a cough ≥ 3 weeks and one half was smear-positive. Finally, 32/167 cases (19.2%) did not declare symptoms and were bacteriologically negative. This percentage was 24.5% (12/49) for cases diagnosed at entry versus 16.9% (20/118) for cases diagnosed by systematic screening (p = 0.25).

X-ray abnormalities observed in the 58 bacteriologically negative cases identified by screening were unilateral in 42/58 cases (72.4%) and bilateral in 16/58 cases (27.6%). With one exception (pleural effusion with pulmonary abnormalities in the lower half of the left lung), these abnormalities were always located in the upper half of the lung(s) and were exclusively apical in 36/58 cases (62.1%). Among these cases with only apical abnormalities, patchy infiltrates were predominant in 26/36 cases (72.2%); in the other 10 cases, fibronodular lesions, small non calcified nodules and, in one case, a single non calcified nodule were observed. In the 21 cases with more extensive abnormalities located in the upper half of the lung(s), a predominance of patchy infiltrates was observed.

## Discussion

The main findings of our follow up study, carried out in a highly endemic prison, are: 1) a sharp decrease in active TB prevalence between the first and the second X-ray systematic screenings conducted at one year interval; 2) a decrease in TB incidence rates of cases identified by passive case-finding between these two years; 3) in inmates screened at entry, the stability of high TB prevalence rates between the first and the second year.

Among the different strategies of TB detection in highly endemic prisons, the detection of symptomatic cases can be done awaiting inmates to come forward spontaneously to the prison dispensaries or be initiated at regular interval by the health service using questionnaires [5], with a better yield of cases but, still, with a limited and slow impact on incidence rates [10]. Our results suggest that, associated with basic control measures and screening at entry, regular periodic X-ray systematic screening (once a year is a common interval) may be a particularly efficient measure as it allows a sharp decrease in incidences and permits the identification of an important number of cases which, as shown by the actual and previous studies [4,13-15], would have been missed by passive case-finding or systematic screening based on symptoms. Our present results are consistent with the predictions obtained by simulating, in our model, the same strategies [10]. In both instances, the prevalence rate of active TB was reduced by around half between the first and the second X-ray screening.

To tentatively explain the important impact of X-ray screening strategies which we observed in this highly endemic prison, we should consider the extensive circulation of MTB strains which we previously demonstrated [16], suggesting that most active TB cases are due to new infections and not to reactivations of previous infections. Control strategies including X-ray screenings may be particularly efficient in this context. 

The predominance of recent exogenous infection by strains circulating in the prison over reactivations may explain, as previously proposed [16], the relatively low frequency of drug-resistant cases observed, in apparent contrast with the high frequency of history of TB treatment. The relatively high cure rate (around 75%) observed in the RJ prison system during the study period and during the years previous to the study [2] may be another explanation.

The diagnosis of TB at an early stage, when contagiousness is lower, particularly important in highly endemic overcrowded and confined environments, can better be achieved by X-ray screening [4,17]. This is consistent with the fact that, in our study, TB cases identified by passive case-finding were more often smear-positive as compared with cases identified by X-ray screeening.

The fact that high TB prevalences among entering inmates, similar to prevalences previously observed at entry in RJ prison system [11], remained stable over the study period reflects the poor conditions of incarceration in the remand centres and other prisons from where entering inmates were transferred. According to international [6-8] and Brazilian recommendations [5,18], the TB screening at entry in prison should be integrated in the mandatory medical examination of entering inmates for the individual benefit of inmates and in order to avoid the import of infectious sources in the prison population.

Given the design of our study, we cannot evaluate the respective impact of screening at entry versus systematic screening on TB prevalence and incidence. However, in highly endemic settings such as the prison we investigated, efficacy of screening at entry on TB prevalence may be limited due to the extensive intra-institutional circulation of strains [16]; in this context, the contribution of new sources of infection from outside to the maintenance of a high level of TB endemicity may be limited. The impact of screening at entry may gain its full efficacy after the level of TB endemicity is decreased by intensive control measures [10].

Given the HIV-seroprevalence we observed among TB cases (2.9%), the overall HIV-seroprevalence in our study population is expected to be low. The impact of the X-ray based screening strategy we have implemented should be assessed as well in prisons where both HIV and TB are highly prevalent. In such prisons, this impact may be different as we expect, among HIV-infected TB cases, a higher percentage of cases resulting from the reactivation of previous infections [19], a lower contagiousness of TB cases [20] and differences in X-ray signs, including TB cases with normal X-ray [21].

The implementation of this strategy of X-ray screening, well accepted by inmates [4,17], may be facilitated, as emphasized by Leung et al. [22], by using a mobile X-ray unit and filmless techniques such as digitalisation or fluoroscopy with a limited exposure to X-ray hazards. Film reading can be performed by adequately trained non-medical personnel which was found by Hoog et al. [23] to be highly sensitive in detecting TB cases. To select subjects who will have a bacteriological examination, the criteria “presence of any radiological abnormality” used in our study should be preferred to the criteria “presence of abnormalities suggestive of TB” in order to limit difficulties of radiological interpretation [13,23]. Thus, X-ray is used as a screening tool to identify TB suspects and not as a diagnostic tool.

The screening procedure we used and the difficulty, for organisational reasons within the prison, to collect early morning sputum samples as recommended [5], may explain the relatively high proportion of bacteriologically-negative cases which did not declare any symptom and were put under TB treatment solely on the basis of chest X-ray findings. In most instances, the X-ray lesions observed in these bacteriologically-negative cases were minimal. The yield of sputum examination is likely to be higher by using the Cepheid Xpert MTB/RIF [14].

If X-ray systematic screening benefits to communities and individuals in general populations, including populations with a high prevalence of TB, remains unclear. However, systematic screening in confined populations at very high TB risk may be cost-efficient as compared with other control strategies as suggested by recent modelling studies [14]. In any case, the implementation of this strategy necessitates an initial investment which, in many low and middle income countries, may be considered as disproportionate given the limited resources dedicated to the prisons health system; in this case, periodic symptom based screening should be implemented. The place of PCR technics in screening algorithms remains to be established [8,14]. Whatever the screening strategy chosen, its implementation would be inappropriate unless good quality passive case-finding and treatment supervision services are provided [8,24].

The current study has two limitations. 1) to measure the impact of systematic X-ray screening on TB prevalence and incidence, a randomized trial would have been much more appropriate, comparing the impact of this strategy to that of alternative interventions [24], but the realization of such a study would be difficult in prison settings for operational and ethical reasons; 2) the implementation of the selected strategy could increase the awareness of TB in the prison and, therefore, the passive case detection rate. But this beneficial collateral effect can be considered as an integral part of any intervention process aimed at increasing the detection of TB.

In any case, in highly endemic prisons, any TB control strategy, even the most intensive, should be associated, to be fully efficient, with drastic reductions of overcrowding and architectural measures aimed at improving ventilation and solar illumination [25,26] in order to limit the extensive strain circulation [16], measures which are actually jointly promoted in Brazil by the Ministries of Justice and Health, and the Global Fund [25,27,28].

## Conclusion

Among the strategies to control TB in prisons such as the prison we investigated, detection at entry as part of the mandatory medical examination on admission and passive case-finding are basic strategies which should be applied, whatever their cost is. As well unquestionable, as an efficient contribution to TB control, are the prevention, diagnostic and treatment of HIV infection - including TB chemoprophylaxis in PPD-reactive HIV infected inmates. Under the condition that its costs in terms of material, staff and functioning does not limit the implementation of the above mentioned measures, the X-ray systematic screening should be considered in highly endemic prisons in order to impact more rapidly on TB prevalence. The impact of environmental interventions which can be implemented without considerable cost [27] is difficult to evaluate, but such interventions should be mandatory in order to comply with international conventions and national laws on prisoner rights [29,30]. Unfortunately, this last strategy and most other strategies mentioned above are not or only partially applied in developing countries in the absence political willingness and will not be fully efficient without a drastic reduction of overcrowding, a key factor [31] which depends for a large part upon the criminal policy and the slow functioning of the justice system as expressed by the high percentages, in most countries, of not sentenced inmates [32].

## Competing interests

The authors declare that they have no competing interests.

## Authors’ contributions

AS and BL drafted the protocol, coordinated the field work, the data entry and management, realized the data analysis and, with MS, drafted the manuscript. VM contributed to the data management, realized the statistical analysis and contributed to manuscript drafting. AB contributed to data collection. GG contributed in the revision of X-rays and revised the manuscript. LBC reviewed the study protocol and contributed to the manuscript. All authors read and approved the final manuscript.

## Pre-publication history

The pre-publication history for this paper can be accessed here:

http://www.biomedcentral.com/1471-2458/13/983/prepub
